# Differential Diagnosis in Musculoskeletal MRIHollenberg GM, Weinberg E, Meyers S. Differential Diagnosis in Musculoskeletal MRI. Thieme Publishers, New York/Stuttgart, 2015; 676 pp, 2043 illustrations, ISBN: 978‐1‐60406‐683‐8. Recommended retail price: AU$279.

**DOI:** 10.1002/jmrs.157

**Published:** 2016-02-22

**Authors:** Debbie Foster

**Affiliations:** ^1^ Auckland Radiology GroupNorthern ClinicAuckland0627New Zealand

## Abstract

Differential Diagnosis in Musculoskeletal MRI is a recent addition to the excellent range of diagnostic imaging references published by Thieme. Although, primarily aimed at clinicians, this is a valuable resource for magnetic resonance imaging (MRI) radiographers who wish to further their knowledge of image interpretation as it covers both common and rare findings. The high quality images and a tabulated format, including discussion on incidence and mechanisms of these disorders, adds to the interest of musculoskeletal MRI.

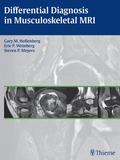

*Differential Diagnosis in Musculoskeletal MRI* is written by eminent radiologists working with the University of Rochester Medical Centre, New York with numerous publications between them on radiological imaging. Published by Thieme, a well respected, award winning, international publisher for more than 125 years, this handsome‐looking book invites investigation. Thieme is known for high quality books and journals in both print and electronic format keeping the medical community up to date with the latest scientific and medical advances.

The book begins with a list of useful medical and magnetic resonance imaging (MRI) abbreviations followed by a brief introduction to MRI for evaluation of musculoskeletal abnormalities. It is then divided roughly into two sections, the first dedicated to lesions of the joints and the second covering bone and soft tissue lesions and tumours. The abnormalities are listed in table format with descriptions of the MRI findings and comments which include clinical presentation, percentage occurrence within age groups and mechanisms of injury. Alongside these are high quality annotated images to illustrate the findings with multiple examples of abnormalities and their appearances. At the end of each section the authors have provided extensive references and suggestions for further reading.

As an experienced MRI radiographer I found this an excellent reference containing high quality paper print and clear images. A list of abbreviations is always a helpful refresher in reading imaging requests in our daily work as well as making sense of the annotated images in this text. However, the table style used in this book took some getting used to and may not suit everyone. I found the more I looked into the book the quicker I found what I was looking for so maybe practice and familiarity are the key to getting the most out of it. The suggested reading and references provide useful background when writing up case studies or further learning and reading lists for continuing professional development (CPD) purposes.

Although this book is aimed at practicing radiologists, fellows, residents and other clinicians, I think it is useful for increasing the breadth of knowledge for all radiographers, particularly those given the opportunity to report MRI. The quality images allow for direct comparison with those on PACS and, along with accompanying descriptions, be able to identify pathology with a greater degree of confidence.

This resource should have a place on the reference bookshelf of any MRI facility particularly those specialising in musculoskeletal studies.

